# Metabarcoding assessment of fungal diversity in brown algae and sponges of Mauritius

**DOI:** 10.3389/fmicb.2022.1003790

**Published:** 2022-10-28

**Authors:** Jessica Mélanie Wong Chin, Daneshwar Puchooa, Theeshan Bahorun, Vidushi S. Neergheen, Aadil Ahmad Aullybux, Girish Beedessee, Nadeem Nazurally, Abdulwahed Fahad Alrefaei, Rajesh Jeewon

**Affiliations:** ^1^Department of Agricultural and Food Science, Faculty of Agriculture, University of Mauritius, Réduit, Mauritius; ^2^Biopharmaceutical Unit, Center for Biomedical and Biomaterials Research (CBBR), University of Mauritius, Réduit, Mauritius; ^3^Department of Biosciences and Ocean Studies, Faculty of Science, University of Mauritius, Réduit, Mauritius; ^4^Department of Biochemistry, University of Cambridge, Cambridge, United Kingdom; ^5^Department of Zoology, College of Science, King Saud University, Riyadh, Saudi Arabia; ^6^Department of Health Sciences, Faculty of Medicine and Health Sciences, University of Mauritius, Réduit, Mauritius

**Keywords:** algae, marine fungi, Mauritius, metagenomics, sponge, ITS regions, phylogeny

## Abstract

Marine fungi are largely associated with second most inhabitants of the marine ecosystem such as sponges and algae. They are important colonizers and play vital ecological roles, such as nutrient cycling, organic matter decomposition, and symbiosis with other organisms. High throughput sequencing methods have been used successfully to reveal unknown fungal communities associated with a number of hosts particularly in the marine environment. However, the diversity of marine fungi associated with sponges and brown algae in Mauritius remains largely unknown. Traditional methods based on culturing do not provide reliable estimate of fungal diversity as only those that are able to grow under laboratory conditions are dominant; in addition, a large proportion of fungi, cultured *in vitro* remain most of the time unidentifiable, given that there are no sporulating structures to be examined morphologically. To overcome these limitations, we employed Illumina sequencing to unravel fungi species present in the sponges, *Iotrochota* sp. and *Biemna* sp. and the brown algae *Turbinaria conoides*, *Sargassum pfeifferae*, and *Sargassum obovatum*, collected from the north of Mauritius. Diversity analyses revealed that *Biemna* sp. had the highest diversity from the sampled sponges with fungi from 24 orders being recovered while from brown algae; *Turbinaria conoides* had the highest diversity with recovery of fungal taxa of the orders *Botryosphaeriales*, *Chaetothyriales*, *Eurotiales*, *Hypocreales*, and *Mucorales* with the latter four orders being common in both sampled algae and sponges. Beta diversity analyses revealed clustering only in the algae, *Turbinaria conoides*, and *Sargassum pfeifferae* and not in the co-occurring sponges, indicating that sampling location did not have much influence on fungal diversity. Our findings provide the first amplicon sequencing based insights of the fungal communities associated with macro-algae and sponges in Mauritius and supplements research on the fungal community existing in the oceans around the world.

## Introduction

Marine organisms live and evolve in a sea of microbes which include bacteria, archaea, microeukaryotes, and viruses. These microorganisms play an important part in the biogeochemical cycles of the marine ecosystems. They can either exist in the planktonic state or live in symbiosis with the animals, plants, and algae of the marine world (Pita et al., 2018). Sponges came into existence approximately 800 million years ago and have formed a close relationship with marine microorganisms, that is, the bacteria, archaea, fungi, and algae (Turner, 2021). The sponge holobiont is a complex relationship that benefits both the host and its associated microorganisms. The latter enhance nutrition, defense mechanisms, immunity, and development of the host while receiving shelter (Suryanarayanan, 2012). Endophytes, often fungi and bacteria, have also been reported from macroalgae around the world. Different microbial biodiversity and bioactive chemical structures have been discovered with immense potentials (Jeewon et al., 2019). An example of a promising anticancer drug in Phase III clinical trial is Plinabulin. It was isolated from the marine fungus *Aspergillus* sp. and is used for the treatment of neutropenia and non-small cell lung cancer (Saeed et al., 2021). Endophytes benefit their host by producing compounds that are useful to growth, protection, and resistance to biotic and abiotic stresses (Nair and Padmavathy, 2014).

Marine fungi are key players in the oceans as they participate actively in the decomposition of organic matter, nutrient cycling and are symbionts to many organisms (Wang et al., 2014). They are taxonomically very diverse which belong to different fungal orders and families, and are also present in different habitats such as deep-sea sediments, hydrothermal vents, sea water, sand, and driftwoods and are associated with a plethora of marine organisms like sponges, corals, sea fans, algae, and sea cucumbers (Godinho et al., 2019). Culture based techniques, using different artificial media, have been employed to study fungal diversity of sponges and algae (Bovio et al., 2018; Sahoo et al., 2021) and these give an indication of some fungal symbionts present and enable the study of bioactive metabolites produced (Bovio et al., 2019; Kamat et al., 2020). Nevertheless, most microorganisms cannot be cultured in the laboratory, and it is often the fast-growing ones that are successfully isolated (Hagestad et al., 2019) and problems associated with traditional methods have been discussed in previous papers (Doilom et al., 2017; Jeewon et al., 2017; Hongsanang et al., 2018). There are very few scientific studies on the marine fungal diversity of Mauritius (Poonyth et al., 1999).

Metagenomics is a resource that can be employed to assess fungal biodiversity and to obtain data about the fungi that cannot be grown under laboratory conditions (Pearman et al., 2019). Many projects have turned to this technique and results are very promising. Eight-hundred and seven fungal operational taxonomic units (OTUs) were obtained by Rämä et al. (2016) on North Atlantic driftwoods, representing a rich biodiversity of Ascomycota and Basidiomycota. More than one fourth of the OTUs were unassigned, pointing the necessity of a wider range of DNA reference data. Garmendia et al. (2021), on the other hand, compared the cultivation-dependent method and the high-throughput sequencing methods (HTS) and concluded that some families of fungi were only obtained by the HTS. The dominant families included Trichocomaceae, Hypocreaceae, Cladosporiaceae, and Mortierellaceae. The in-depth investigation of the mycobiome will enable better understanding of the ecological functions of these symbionts. The orders and phyla of marine fungi associated with the sponges and algae can be identified and their functional roles assigned (Moreno-Pino et al., 2020). However, this method requires knowledge in bioinformatics, proper planning of the experiment, and appropriate standards to ensure reproducibility (Escobar-Zepeda et al., 2015).

To the best of our knowledge, no sequencing approach has been applied to survey the fungal diversity present in the brown algae and sponges from Mauritius waters. These samples were chosen as they support a high diversity of fungi (Suryanarayanan et al., 2010; Bovio et al., 2018). To this end, we employed ITS-targeted amplicon sequencing to survey the diversity of fungal communities of these organisms.

## Materials and methods

### Sample collection

**Figure 1:**
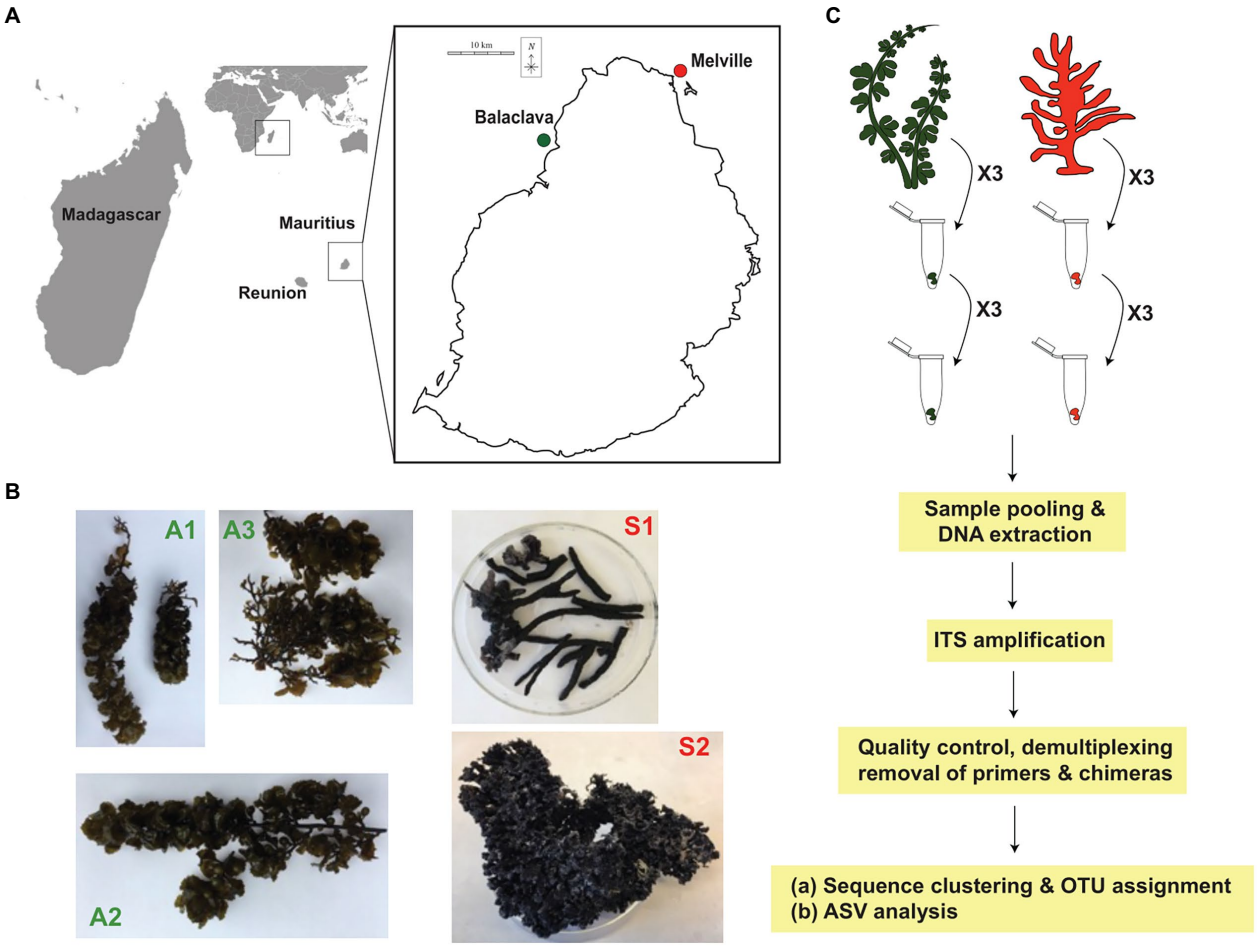
Samples collection in Mauritius and experimental design. **(A)** Red and green dots represent sites where sponge and algal samples were collected, respectively. **(B)** Brown algae (A1: *Turbinaria conoides*, A2: *Sargassum pfeifferae*, A3: *Sargassum obovatum*), and sponges (S1: *Iotrochota* sp., S2: *Biemna* sp.) used in extraction of metagenomic DNA. **(C)** Experimental design and pipeline for data analysis.

The brown algae, *Turbinaria conoides* (A1), *Sargassum pfeifferae* (A2), and *Sargassum obovatum* (A3) were collected in August 2020 (average temperature around 20°C) in Balaclava, Mauritius (coordinate: 20°08′30′′ S, 57°51′65′′ E) at a depth of 1 m. The algae were identified according to Guiry and Guiry (2020). The sponge samples, *Iotrochota* sp. (S1) and *Biemna* sp. (S2) were collected in August 2018 in Melville, Mauritius (coordinate: 20°1′34′′ S; 57°42′8′′ E; Figures 1A,B) at a depth of 1 m and were identified according to the World Porifera Database (https://www.marinespecies.org/porifera/; Van Soest et al., 2020). Samples were placed in Ziploc® bags with sea water and were transported on ice. The sponges and brown algae were then washed in sterile sea water to remove any debris, surface sterilized in 70% ethanol for 30 s followed by three rinses in autoclaved sea water, and frozen at −20°C for DNA extraction.

### DNA extraction and PCR amplification

The samples were crushed in liquid nitrogen and DNA extraction was carried out using the Norgen Biotek Plant/Fungi DNA isolation kit (Norgen Biotek Corp, Cat. 26200) according to the manufacturer’s instructions. Three biological replicates were used for each sample and three technical replicates were included as shown in Figure 1C. The DNA samples were sent to Novogene Co., Ltd. (Singapore) for amplification of the ITS regions (ITS1) and sequencing. PCR reactions were conducted using Phusion ® High-fidelity PCR Master Mix (New England Biolabs), with the primers ITS5-1737F (GGAAGTAAAAGTCGTAACAAGG) and ITS2-2043R (GCTGCGTTCTTCATCGATGC; White et al., 1990). PCR amplification was performed using the following conditions: 95°C for 120 s; 32 cycles of 94°C for 30s, 55°C for 30s, 72°C for 45 s; and 72°C for 10 min (Bellemain et al., 2010). The negative control consisted of nuclease-free water instead of the sample DNA. PCR products were checked on 2% agarose gel and quantity and purity was assessed by Nanodrop and Qubit 2.0. Products between 400–450 bp were mixed at equal density ratios and purified using the Qiagen Gel Extraction Kit (Qiagen, Germany). Sequencing was performed using the Illumina NovaSeq PE250. The libraries were generated with NEBNext® UltraTM DNA Library Prep Kit for Illumina (New England Biolabs, Cat. E7370L).

### Sequence analysis of ITS amplicon community profiling

Paired-end reads were first demultiplexed by assigning them to their corresponding samples according to their unique barcodes. They were then truncated by cutting the barcode and primer sequences before being merged using FLASH (version 1.2.7; Magoč and Salzberg, 2011). Quality control was subsequently performed on the merged sequences using the default parameters in QIIME (version 1.7.0; Caporaso et al., 2010), to remove low quality reads and those containing ambiguous bases so as to obtain high quality clean tags (Bais et al., 2006). These were compared to sequences in the UNITE database (Gold database, http://drive5.com/uchime/uchime_download.html) using the UCHIME algorithm (UCHIME Algorithm, http://www.drive5.com/usearch/manual/uchime_algo.html) to identify and remove chimeric sequences as well as singletons and host sequences. The resulting sequences were clustered into operational taxonomic units (OTUs) at 97% similarity using UPARSE (version 7.0.1001; Edgar, 2013) before assigning taxonomy by BLAST searches against the UNITE database.^1^ The metagenome sequences were deposited in the NCBI BioSample database with accession numbers SAMN18168348–SAMN18168352, and the project was registered in NCBI Sequence Read Archive (SRA) under the BioProject accession number PRJNA707034.

### Diversity metrics and statistical analysis

The community richness, diversity of the brown algae, and sponge samples as well as the sequence coverage were determined using alpha diversity indices. The Chao1 estimator, Shannon index, and the Good’s coverage, estimated in QIIME were used, and displayed with the ggplot2 package (Wickham, 2016) of R (version 2.15.3 R core team 2020; Nenadic and Greenacre, 2020). T-test and Wilcoxon Rank Sum tests were performed to analyze the significant differences between the samples. Beta diversity was used to assess the differences in fungal composition between the samples based on Unweighted Unifrac distances. The Analysis of MOlecular VAriance (AMOVA) test was used to assess significant difference between samples. Similarities or dissimilarities between the samples were visualized on a Principal Coordinate Analysis (PCoA) plot in R.

### ASV workflow data analysis

In addition to OTU clustering, the amplicon sequence variant (ASV) approach was also employed. DADA2 inference algorithm was used on barcode and primer-free sequences were used to correct for errors and create ASVs for the fungal communities (Callahan et al., 2016). The error model algorithm of DADA2 was applied, followed by dereplication, sample inference, merging of paired-end reads, and chimera removal in order to generate a sequence table of abundance per sample. Taxonomy was assigned based on the curated UNITE database. IdTaxa *via* the DECIPHER package (Murali et al., 2018) was used for taxonomic classification. Community-based analyses were conducted by looking at the ASV abundance in all the samples. To assess within sample diversity (i.e., alpha), the phyloseq package (McMurdie and Holmes, 2013) was used. To assess in between sample diversity (i.e., beta), dissimilarities were computed using the ordinate function in phyloseq and visualized using principal coordinates analysis.

## Results

### Composition of fungal communities

**Figure 2:**
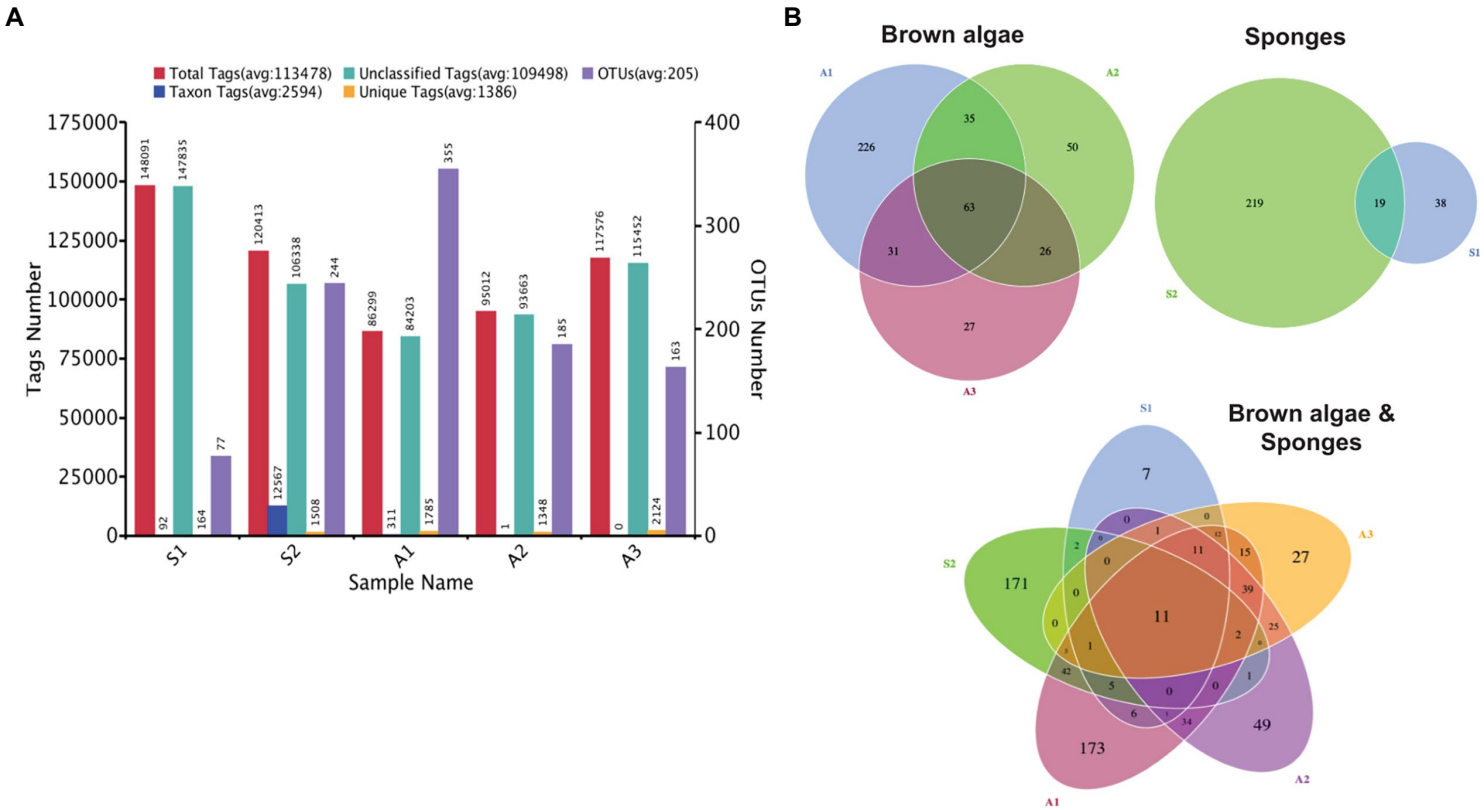
Number of tags and operational taxonomic units (OTUs). **(A)** Summary of the number of tags; total tags, taxon tags, unclassified tags, unique tags, and OTUs of sponge and algae samples. **(B)** Venn diagrams of shared and unique OTUs between the co-occurring brown algae (top left), sponges (top right), and the five samples (down center) included in this study. Number of OTUs is indicated within each shape.

After the sequencing of the fungal ITS regions, a total of 755,822 reads were generated. From these, only 567,391 reads were of high quality after removing low-quality reads and filtering chimeras. The sample A1 had the smallest number of bases (26,878,973) whereas the sample S1 had the largest number of bases (49,872,062). The average number of bases for the five samples was 36,479,404. The average length of the effective tags ranged from 267 to 360 bp. A detailed summary of the number of total tags, the taxon, and unclassified ones, the number of unique tags as well as the number of OTUs in the five samples is provided in Figure 2A; Table 1. A1 had the smallest number of reads and the highest number of OTUs. For the co-occurring brown algae, 63 OTUs were shared among the three macroalgae and this could be indicative of a core microbiome for macroalgal species. When comparing two species of brown algae, 35 OTUs were common between A1 and A2, 31 OTUs between A1 and A3, and 26 OTUs were shared between A2 and A3. In the case of sponges, only 19 OTUs were common between them (Figure 2B).

**Table 1:** Total number of tags, taxon tags, unclassified tags, unique tags, and OTUs.

Sample	Total Tags	Taxon Tags	Unclassified Tags	Unique Tags	OTUs
S1	148,091	92	147,835	164	77
S2	120,413	12,567	106,338	1,508	244
A1	86,299	311	84,203	1,785	355
A2	95,012	1	93,663	1,348	185
A3	117,576	0	115,452	2,124	163

### Community composition at order and genera level

**Figure 3:**
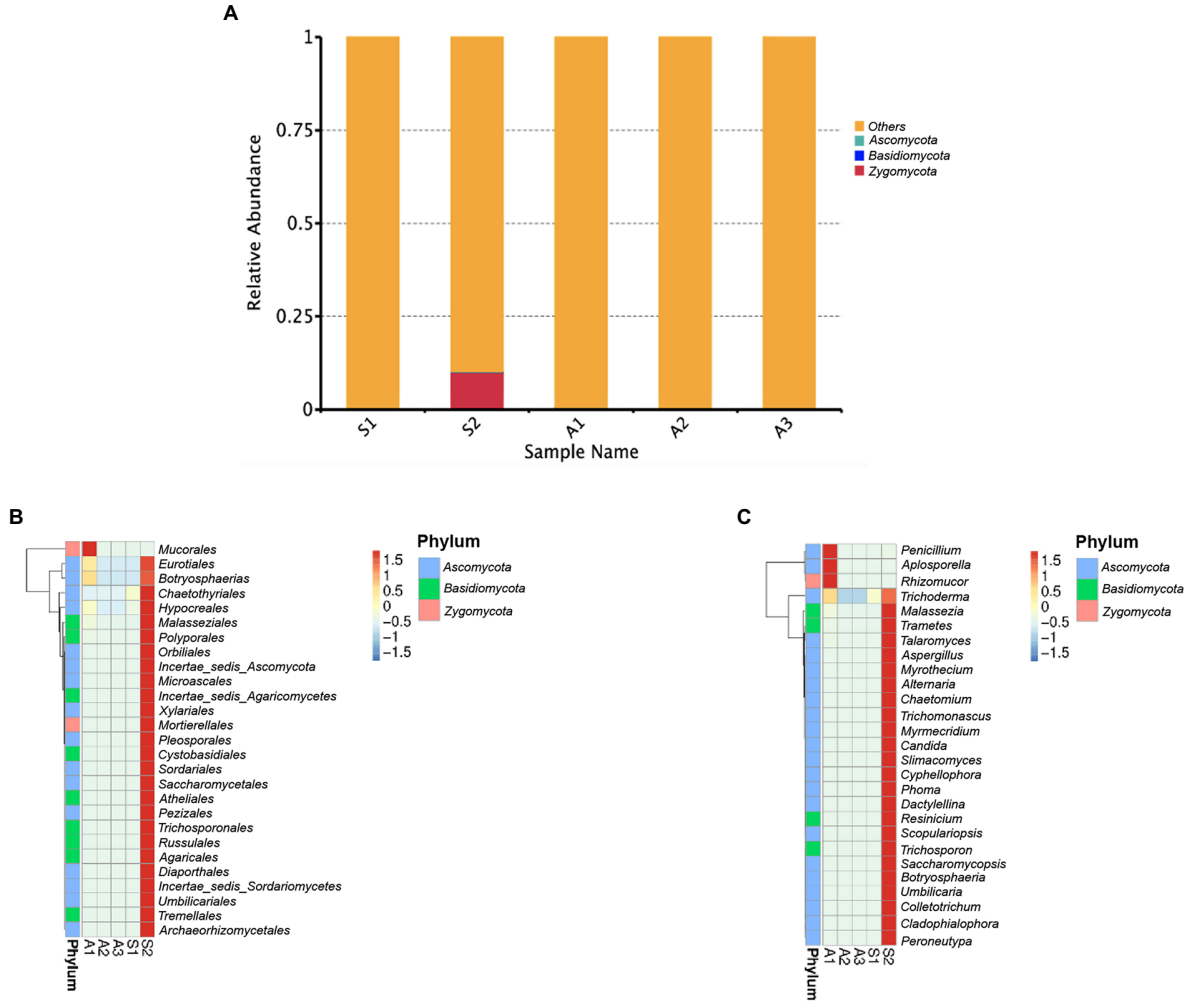
Composition of fungal Community. **(A)** Relative abundance of fungal phyla in each sample. **(B,C)** Heat map of orders and genera of fungi found in the brown algae and sponges based on OTU data.

Only fungal taxa of the *Ascomycota* and *Basidiomycota* were recovered from sponge S2 (Figure 3A; Supplementary Figure 1A). The relative abundance of *Ascomycota* was 0.0995% and *Basidiomycota* was of 0.00118%. Sponge S2 harbored more diverse and abundant orders as compared to sponge S1 which did not have a high abundance of identified fungi. Sponge S1 was colonized by fungal taxa of the orders *Botryosphaeriales*, *Chaetothyriales, Eurotiales*, and *Hypocreales*, which were common to sponge S2, with the latter also containing the 24 orders shown except for *Mucorales* (Figure 3B; Supplementary Figures 1B,C). At the genus level, only *Trichoderma* was common to the two sponges. Twenty-four genera, namely *Alternaria, Aspergillus*, *Botryosphaeria*, *Candida*, *Chaetomium Cladophialophora, Colletotrichum, Cyphellophora*, *Dactylellina*, *Malassezia*, *Myrmecridium Myrothecium*, *Peromneutypa, Phoma*, *Resinicium*, *Saccharomycopsis*, *Scopulariopsis, Slimacomyces, Talaromyces*, *Trametes*, *Trichoderma, Trichomonascus*, *Trichosporon*, and *Umbilicaria* were obtained from the sponge S2 (Figure 3C).

For the brown algae, A1 had the highest diversity and abundance of fungal orders namely *Botryosphaeriales*, *Chaetothyriales, Eurotiales*, *Hypocreales, Malasseziales*, and *Mucorales*. In contrast, algae A2 and A3 contained lower abundances of *Botryosphaeriales*, *Chaetothyriales, Eurotiales*, and *Hypocreales*. Common orders that were detected in the three algae included *Botryosphaeriales, Chaetothyriales, Eurotiales*, and *Hypocreales* (Figure 3B). Regarding fungal genera, all the three algae harbored fungal taxa of the genus *Trichoderma*, with algae A1 also being colonized by the genera *Aplosporella*, *Malassezia, Penicillium*, and *Rhizomucor* (Figure 3C).

### Statistical analyses

**Figure 4:**
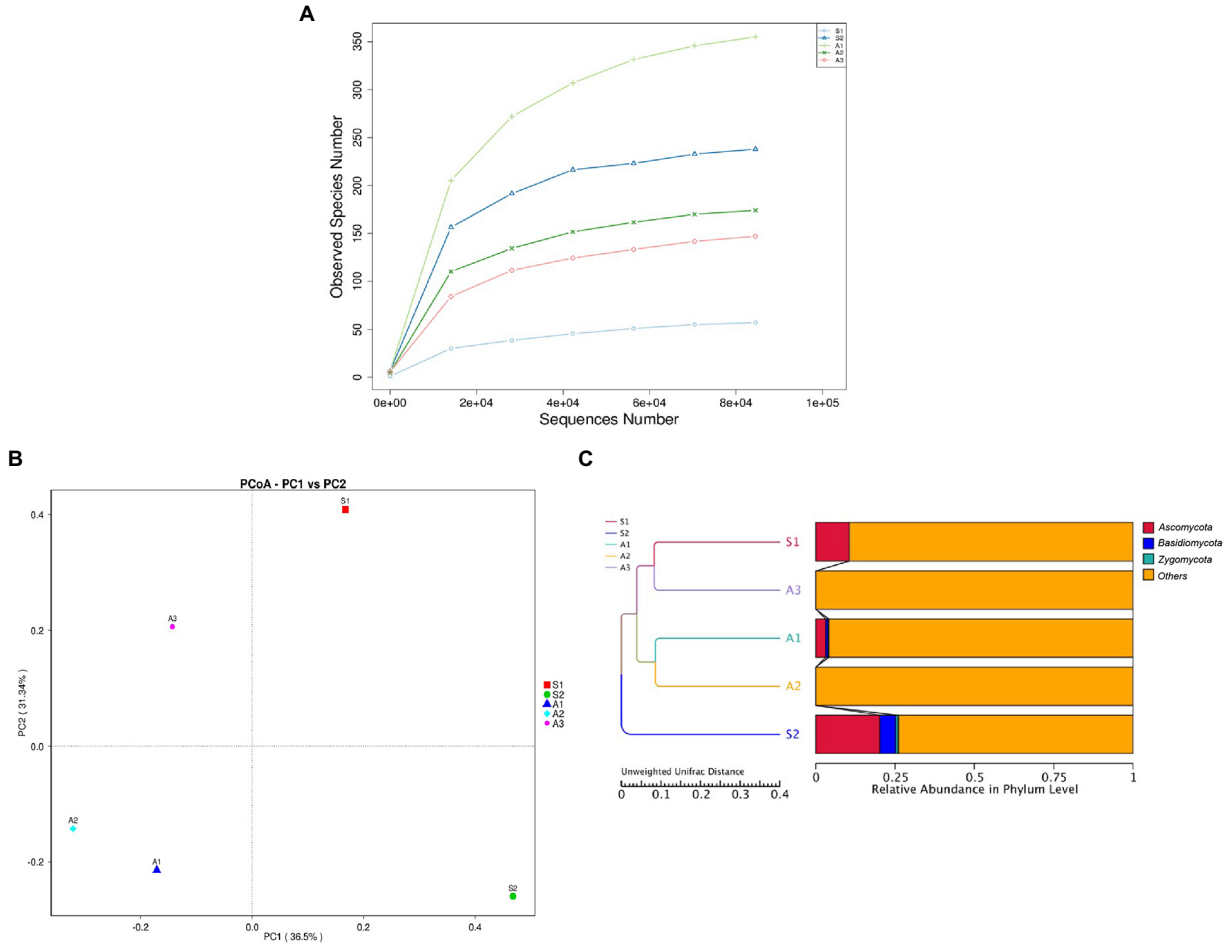
Alpha diversity and beta diversity analysis. **(A)** Rarefaction curve of fungal OTUs in sponges and algae **(B)** Principal co-ordinate analysis (PCoA) analysis of relative fungal abundance. **(C)** UPGMA cluster tree based on Unweighted Unifrac distance.

The alpha diversity reflects the fungal community diversity within a sample. It indicates the richness and evenness of fungal communities found within each sample. Three indices were used in this study, namely the Shannon diversity index, the Chao1index, and the Good’s coverage (Table 2; Supplementary Figure 2A). The Shannon diversity index and the Chao1 were in accordance with each other and indicated a significantly lower fungal diversity in sponge S1 as compared to S2, A3, A2, and S1 (*p* < 0.05). The Good’s coverage of all the samples were 100%, indicating that the sequencing depths were sufficient to evaluate fungal diversity in the samples. This was supported by the rarefaction curve (Figure 4A; Supplementary Figure 2B) where the curves show a tendency to become flatter.

**Table 2:** Alpha diversity indices results.

Sample Name	Observed Species	Shannon	Chao1	Goods coverage
S1	57	0.146	65.750	1.000
S2	238	3.976	249.607	1.000
A1	355	4.118	363.470	1.000
A2	174	2.917	179.135	1.000
A3	147	3.055	157.969	1.000

The beta diversity analysis was used to measure the differences between community composition of the different samples. From the AMOVA test, there were no significant differences between the samples (*p* > 0.05). Based on relative fungal abundance, the samples collected from the same region did form a close group, with A1 and A2 being particularly closer. The two PCoA axes accounted for 36.5 and 31.3% of the observed variations, respectively, (Figure 4B). Similar results were observed in the phylogenetic-based clustering of samples, where the two brown algae A1 and A2, obtained from the same environment, clustered together, thereby showing similarities in the fungal community composition. Macroalgae A3 was also obtained from the same environment but it clustered with sponge S1, which was collected at another location. Sponge S2 did not cluster with the rest of the samples (Figure 4C).

## Discussion

Fungal biodiversity in Mauritius waters has not been profoundly studied. Identity-based OTU clustering is the most commonly-used approach to reveal fungal diversity from different environments, and our data were comparable in both the OTU and ASV approaches. Therefore, OTU data were used to discuss the results. In the sponge *Iotrochota* sp. (S1), fungal taxa from only three orders were detected, namely *Botryosphaeriales*, *Chaetothyriales, Eurotiales*, *and Hypocreales.* These were found at a relatively low abundance. This is quite unusual as most genomic analyses of sponges have revealed a high abundance and diversity of fungal communities (Moreno-Pino et al., 2020). Given that it was co-occurring in the same environment as the sponge *Biemna* sp. (S2), which was colonized by numerous fungi, a wider range of orders was expected. Nevertheless, there are sponges which have low-microbial abundance, that is, they have a microbial biomass, which is less diverse and single-clade dominant (Fuerst, 2015). He et al. (2014) found only one OTU to be present in the sponges *Cinachyrella australiensis* and *Haliclona mediterranea* from the South China Sea. In the same study, 1,122 OTUs were discovered from the sponge *Iotrochota* sp., which represented much more than the 57 OTUs found in our study. Fungal colonization depends on environmental conditions as well as the host and therefore, it can be variable for different sponges. Marine fungi have shown to adapt and evolve differently based on the environmental conditions in which they live. These conditions exert a selective pressure on the microorganisms. In addition, the sponge *Iotrochota* sp. is known to produce the halogenated alkaloids purpuroines A–J, which might have antifungal properties (Shen et al., 2012). The low abundance of fungi in the sponge S1 might be due to the secondary metabolites produced by this sponge.

In this study, the sponge *Biemna* sp. had the highest fungal diversity with *Ascomycota* and *Basidiomycota* being the two most identified phyla. Nguyen and Thomas (2018) also found that *Ascomycota*, *Basidiomycota*, and *Glomeromycota* colonized the three sponges *Cymbastela concentrica*, *Scopalina* sp., and *Tedania ahelans* from Australia. This confirms that these phyla are common colonizers of marine sponges (Paz et al., 2010; Jin et al., 2014; Naim et al., 2017). Furthermore, fungal taxa from orders of the *Agaricales*, *Diaporthales, Eurotiales, Hypocreales*, *Malasseziales*, *Pleosporales*, and *Xylariales* were also recovered, similar to the ones found in the sponge *Biemna* sp. Nineteen OTUs were shared between sponge S1 and S2. The orders *Botryosphaeriales*, *Chaetothyriales*, *and Eurotiales* were found in both sponges and might be indicative of a core microbiome. These orders have been found in different sponges around the world and might occupy important functions in the sponge holobiont (Yu et al., 2013; He et al., 2014; Bovio et al., 2019; Karthik and Li, 2019). The genera *Aspergillus* and *Penicillium* have often been recovered from marine sponges and macroalgae (Venkatachalam et al., 2015; Yang et al., 2018; Moreno-Pino et al., 2020; Sibero et al., 2021) and our study reports *Aspergillus* in the sponge *Biemna* sp. and that of *Penicillium* in the brown algae *Turbinaria conoides*. It can be noted that orders of yeasts have also been identified in the sponge S2. This is not uncommon as Naim et al. (2017) obtained reads that belonged to the yeast orders *Malasseziales*, *Saccharomycetales*, *Cystofilobasidiales*, and *Tremellales*. Maldonado et al. (2005) also reported that in sponges, endosymbiotic yeasts are maternally transmitted through the oocytes to the fertilized egg. This indicates that yeasts, along with filamentous fungi inhabit the sponges.

Regarding the algal fungal community, common orders that were shared between the three macroalgae were *Botryosphaeriales, Eurotiales*, and *Hypocrelaes*. *Eurotiales* and *Hypocreales* have also been recovered from macroalgae by Abdel-Gawad et al. (2014) while Wainwright et al. (2019) recovered these orders from Illumina sequencing of *Sargassum ilicifolium*. Yurchenko et al. (2019) isolated an algal-derived fungus *Penicillium* sp. indicating that this genus is an algal endophyte. The order *Botryosphaeriales* was found in the marine environment and was isolated as an endophyte of the red algae *Bostrychia radicans* (De Jesus et al., 2017). Therefore, these three orders could represent the core microbiome of macroalgae. As endophytes, they have important roles in their hosts, participating in protection, growth and resistance (Morelli et al., 2020; Ogbe et al., 2020). The genus *Trichoderma* was recovered in the three algae in this study. Song et al. (2019) also isolated a marine algal endophyte *Trichoderma asperellum* with interesting bioactive properties. Marine endophytic *Trichoderma* can inhibit marine-derived bacteria and phytoplankton (Zou et al., 2019; Liu et al., 2020). Our results suggest that these three algae contain endophytes that could be further studied for the production of bioactive secondary metabolites. The high abundance of sequences with unassigned taxonomy in this study was not unexpected. Previous studies on the green macroalgae *Caulerpa lentillifera* revealed five eukaryotic phyla along with unclassified eukaryotes based on ITS sequencing. The unclassified sequences accounted for 83.6% of all the sequences at the genera level (Liang et al., 2019). The metabarcoding analyses of Xu et al. (2019) of the deep-sea hadal sediments also yielded 80% of total OTUs which were not assigned to any fungal division. Wainwright et al. (2019) also reported their inability to assign taxonomy to many sequences obtained from the brown algae *Sargassum ilicifolium*. They concluded that the marine environment has various unknown fungi that are not referenced in the databases. Inability to assign taxonomy is a major problem in marine fungi high throughput sequencing studies. This arises due to the lack of representation in the public databases. As most fungal research focus on terrestrial environments, sequence data of fungi that live solely in the marine world are minim (Lee et al., 2019; Wainwright et al., 2019; Xu et al., 2019).

The beta analyses as well as the phylogenetic relationship revealed that only the two algae, *Turbinaria conoides* (A1) and *Sargassum pfeifferae* (A2) clustered relatively close to each other, reflecting the fact that these algae were co-occurring in the same environment and therefore, it is evident that some fungi would be present in both algae. Although the sponges were collected from the same location, they did not cluster together but they did share common OTUs, indicating that there are fungi from their environment that live in association with them. According to Nguyen and Thomas (2018), the sponges *T. anhelans* and *Scopalina* sp. were significantly different in fungal communities, suggesting that the sponge exert different selective pressures on their fungal communities. A similar observation was made by He et al. (2014), where co-occurring sponges *Haliclona fascigera* and *Ircinia* sp. did not cluster together too. Gao et al. (2008) also supported the theory that eukaryotic diversity of sponges has no correlation with the sampling location and this might explain why sponge S1 did not cluster with sponge S2.

The culture-independent method used in this study showed higher fungal diversity compared to a previous traditional microbiological study from our research group that involved the same sponge and algal species (Wong Chin et al., 2021). The marine ecosystem provides abundant opportunities to discover novel fungi that can advance our understanding of fungal diversity and ecology while expanding the current repertoire of organisms from the Mauritius waters with the potential to contribute to drug discovery.

## Conclusion

This study demonstrated that many orders of marine fungi inhabit sponges and algae in Mauritius. Various uncultured and yet-to-be discovered fungi have high abundance in the samples in this study. The orders and genera that were described have been isolated from marine environments around the world and are not uncommon. This indicates that they are sponge and algal generalists. The samples from the same locations did not show specific clustering, suggesting that the host might exert more pressure on the fungal diversity as compared to the environment. This NGS-based cultivation-independent method is a powerful tool to uncover fungal communities but further studies are needed to shed light on the function of these fungi in the sponges and algae of Mauritius and how they contribute and interact in their host’s survival and protection.

## Data availability statement

The original contributions presented in the study are included in the article/Supplementary material, further inquiries can be directed to the corresponding author.

## Author contributions

RJ and DP designed the study and supervised the work. JMWC is a PhD student working on the project and did the sample collections, laboratory work, and analyses. AAA and GB provided assistance with DNA analyses, figures, and writing up. TB, VN, NN, and AFA also contributed to laboratory assistance and editing of the article. All authors contributed to the writing up of the manuscript. All authors contributed to the article and approved the submitted version.

## Funding

This study was supported by the Higher Education Commission (HEC; MPhil/PhD Scholarship).

## Conflict of interest

The authors declare that the research was conducted in the absence of any commercial or financial relationships that could be construed as a potential conflict of interest.

## Publisher’s note

All claims expressed in this article are solely those of the authors and do not necessarily represent those of their affiliated organizations, or those of the publisher, the editors and the reviewers. Any product that may be evaluated in this article, or claim that may be made by its manufacturer, is not guaranteed or endorsed by the publisher.

## Footnotes

^1^ https://unite.ut.ee/

## Supplementary material

Supplementary Figure 1 Community composition. **(A)** Taxonomic summary at phylum level in all the samples. **(B)** Boxplot showing percentage of ITS gene copies recovered in all the samples. **(C)** Boxplot showing fungal diversity in sponge (red dots) and algae (green dots) separately.

Supplementary Figure 2 Alpha and beta diversity analysis. **(A)** Chao1 richness estimates and Shannon diversity values in all the samples combined and types. **(B)** Rarefaction curve describing fungal richness observed from sponges and algae sampled.

## References

[ref1] Abdel-Gawad K. M. Hifney A. F. Issa A. A. Gomaa M. (2014). Spatio-temporal, environmental factors, and shape culturable-epibiotic fungi of seaweeds in the Red Sea, Egypt. Hydrobiologia 740, 37–49. doi: 10.1007/s10750-014-1935-0

[ref2] Bais H. P. Weir T. L. Perry L. G. Gilroy S. Vivanco J. M. (2006). The roles of root exudates in rhizosphere interactions with plants and other organisms. Annu. Rev. Plant Biol. 57, 233–266. doi: 10.1146/annurev.arplant.57.032905.105159, PMID: 16669762

[ref3] Bellemain E. Carlsen T. Brochmann C. Coissac E. Taberlet P. Kauserud H. (2010). ITS as an environmental DNA barcode for fungi: an *in silico* approach reveals potential PCR biases. BMC Microbiol. 10:189. doi: 10.1186/1471-2180-10-189, PMID: 20618939 PMC2909996

[ref4] Bovio E. Garzoli L. Poli A. Luganini A. Villa P. Musumeci R. (2019). Marine fungi from the sponge *Grantia compressa*: biodiversity, Chemodiversity, and biotechnological potential. Mar. Drugs 17:220. doi: 10.3390/md17040220, PMID: 30978942 PMC6520677

[ref5] Bovio E. Garzoli L. Poli A. Prigione V. Firsova D. McCormack G. P. (2018). The culturable mycobiota associated with three Atlantic sponges, including two new species: *Thelebolus balaustiformis* and *T. spongiae*. Fungal System. Evol. 1, 141–167. doi: 10.3114/fuse.2018.01.07, PMID: 32490365 PMC7259239

[ref6] Callahan B. J. McMurdie P. J. Rosen M. J. Han A. W. Johnson A. J. A. Holmes S. P. (2016). DADA2: high-resolution sample inference from Illumina amplicon data. Nat. Methods 13, 581–583. doi: 10.1038/nmeth.3869, PMID: 27214047 PMC4927377

[ref7] Caporaso J. G. Kuczynski J. Stombaugh J. Bittinger K. Bushman F. D. Costello E. K. (2010). QIIME allows analysis of high-throughput community sequencing data. Nat. Methods 7, 335–336. doi: 10.1038/nmeth.f.303, PMID: 20383131 PMC3156573

[ref8] De Jesus H. C. R. Jeller A. H. Debonsi H. M. Alves P. B. Porto A. L. M. (2017). Multiple monohydroxylation products from rac-camphor by marine fungus *Botryosphaeria* sp. isolated from marine alga *Bostrychia radicans*. J. Braz. Chem. Soc. 28, 498–504. doi: 10.21577/0103-5053.20160262

[ref9] Doilom M. Manawasinghe I. S. Jeewon R. Jayawardena R. S. Tibpromma S. Hongsanan S. (2017). Can ITS sequence data identify fungal endophytes from cultures? A case study from *Rhizophora apiculata*. Mycosphere 8, 1869–1892. doi: 10.5943/mycosphere/8/10/11

[ref10] Edgar R. C. (2013). UPARSE: highly accurate OTU sequences from microbial amplicon reads. Nat. Methods 10, 996–998. doi: 10.1038/nmeth.2604, PMID: 23955772

[ref11] Escobar-Zepeda A. de León A. V. P. Sanchez-Flores A. (2015). The road to metagenomics: from microbiology to DNA sequencing technologies and bioinformatics. Front. Genet. 6:348. doi: 10.3389/fgene.2015.00348 26734060 PMC4681832

[ref12] Fuerst J. A. (2015). Diversity and biotechnological potential of microorganisms associated with marine sponges. Appl. Microbiol. Biotechnol. 98, 7331–7347. doi: 10.1007/s00253-014-5861-x 25005058

[ref13] Gao Z. Li B. Zheng C. Wang G. (2008). Molecular detection of fungal communities in the Hawaiian marine sponges *Suberites zeteki* and *Mycale armata*. Appl. Environ. Microbiol. 74, 6091–6101. doi: 10.1128/AEM.01315-08 18676706 PMC2565983

[ref14] Garmendia G. Alvarez A. Villarreal R. Martínez-Silveira A. Wisniewski M. Vero S. (2021). Fungal diversity in the coastal waters of King George Island (maritime Antarctica). World J. Microbiol. Biotechnol. 37:142. doi: 10.1007/s11274-021-03112-4, PMID: 34322842

[ref15] Godinho V. M. de Paula M. T. R. Silva D. A. S. Paresque K. Martins A. P. Colepicolo P. (2019). Diversity and distribution of hidden cultivable fungi associated with marine animals of Antarctica. Fungal Biol. 123, 507–516. doi: 10.1016/j.funbio.2019.05.001, PMID: 31196520

[ref16] Guiry M.D. Guiry G. M. (2020). AlgaeBase. Available at: https://www.algaebase.org (Accessed March, 2020).

[ref17] Hagestad O. C. Andersen J. H. Altermark B. Hansen E. Rämä T. (2019). Cultivable marine fungi form the Arctic archipelago of Svalbard and their antibacterial activity. Mycology 11, 230–242. doi: 10.1080/21501203.2019.1708492, PMID: 33062384 PMC7534220

[ref18] He L. Liu F. Karuppiah V. Ren Y. Li Z. (2014). Comparisons of the fungal and protistan communities among different marine sponge holobionts by pyrosequencing. Microb. Ecol. 67, 951–961. doi: 10.1007/s00248-014-0393-6, PMID: 24577740

[ref19] Hongsanang S. Jeewon R. Purahong W. Xie N. Liu J.-K. Jayawardena R. S. (2018). Can we use environmental DNA as holotypes? Fungal Divers. 92, 1–30. doi: 10.1007/s13225-018-0404-x

[ref20] Jeewon R. Luckun A. B. Bhoyroo V. Sadeer N. B. Mahomoodally M. F. Rampadarath S. (2019). “Pharmaceutical potential of marine fungal endophytes” in Endophytes and Secondary Metabolites. ed. Jha S. (Cham: Reference Series in Phytochemistry: Springer), 1–23.

[ref21] Jeewon R. Wanasinghe D. N. Rampadaruth S. Puchooa D. Zhou L. G. Liu A. R. (2017). Nomenclatural and identification pitfalls of endophytic mycota based on DNA sequence analyses of ribosomal and protein genes phylogenetic markers: a taxonomic dead end? Mycosphere 8, 1802–1817. doi: 10.5943/mycosphere/8/10/7

[ref22] Jin L. Liu F. Sun W. Zhang F. Karuppiah V. Li Z. (2014). Pezizomycotina dominates the fungal communities of South China Sea sponges *Theonella swinhoei* and *Xestospongia testudinaria*. FEMS Microbiol. Ecol. 90, 935–945. doi: 10.1111/1574-6941.12446, PMID: 25348120

[ref23] Kamat S. Kumari M. Taritla S. Jayabaskaran C. (2020). Endophytic fungi of marine alga from Konkan coast, India-a rich source of bioactive material. Front. Mar. Sci. 7:31. doi: 10.3389/fmars.2020.00031

[ref24] Karthik L. Li Z. (2019). “Marine enzymes from microbial symbionts of sponges and corals” in Symbiotic Microbiomes of Coral Reefs and Sponges and Corals. ed. Li Z. (Netherlands: Springer Nature BV), 527–542.

[ref25] Lee N. L. Y. Huang D. Quek Z. B. R. Lee J. N. Wainwright B. J. (2019). Mangrove-associated fungal communities are differentiated by geographic location and host structure. Front. Microbiol. 10:2456. doi: 10.3389/fmicb.2019.02456, PMID: 31736902 PMC6831645

[ref26] Liang Z. Liu F. Wang W. Zhang P. Sun X. Wang F. (2019). High-throughput sequencing revealed differences of microbial community structure and diversity between healthy and diseased *Caulerpa lentillifera*. BMC Microbiol. 19:225. doi: 10.1186/s12866-019-1605-5, PMID: 31615401 PMC6794861

[ref27] Liu X. H. Hou X. L. Song Y. P. Wang B. G. Ji N. Y. (2020). Cyclonerane sesquiterpenes and an isocoumarin derivative from the marine-alga-endophytic fungus *Trichoderma citrinoviride* A-WH-20-3. Fitoterapia 141:104469. doi: 10.1016/j.fitote.2020.104469, PMID: 31917301

[ref28] Magoč T. Salzberg S. L. (2011). FLASH: fast length adjustment of short reads to improve genome assemblies. Bioinformatics 27, 2957–2963. doi: 10.1093/bioinformatics/btr507, PMID: 21903629 PMC3198573

[ref29] Maldonado M. Cortadellas N. Trillas M. I. Rützler K. (2005). Endosymbiotic yeast maternally transmitted in a marine sponge. Biol. Bull. 209, 94–106. doi: 10.2307/3593127, PMID: 16260769

[ref30] McMurdie P. J. Holmes S. (2013). Phyloseq: an R package for reproducible interactive analysis and graphics of microbiome census data. PLoS One 8:e61217. doi: 10.1371/journal.pone.0061217, PMID: 23630581 PMC3632530

[ref31] Morelli M. Bahar O. Papadopoulou K. K. Hopkins D. L. Obradovic A. (2020). Editorial: role of endophytes in plant health and defense against pathogens. Front. Plant Sci. 11:1312. doi: 10.3389/fpls.2020.01312, PMID: 32983202 PMC7479191

[ref32] Moreno-Pino M. Cristi A. Gillooly J. F. Trefault N. (2020). Characterizing the microbiomes of Antarctic sponges: a functional metagenomics approach. Sci. Rep. 10:645. doi: 10.1038/s41598-020-57464-2, PMID: 31959785 PMC6971038

[ref33] Murali A. Bhargava A. Wright E. S. (2018). IDTAXA: a novel approach for accurate taxonomic classification of microbiome sequences. Microbiome 6:140. doi: 10.1186/s40168-018-0521-5, PMID: 30092815 PMC6085705

[ref34] Naim M. A. Smidt H. Sipkema D. (2017). Fungi found in the Mediterranean and North Sea sponges: how specific are they? PeerJ. 5:e3722. doi: 10.7717/peerj.3722, PMID: 28894639 PMC5591636

[ref35] Nair D. Padmavathy S. (2014). Impact of endophytic microorganisms on plants, environment and humans. Sci. World J. 2014, 1–11. doi: 10.1155/2014/250693, PMID: 24587715 PMC3920680

[ref36] Nenadic O. Greenacre M. (2020). Correspondence analysis in R, with two-and three-dimensional graphics: The ca package. Available at: http://resolver.sub.uni-goettingen.de/purl?gs-1/5892 (Accessed March, 2021).

[ref37] Nguyen M. T. H. D. Thomas T. (2018). Diversity, host-specificity and stability of sponge-associated fungal communities of co-occurring sponges. PeerJ. 6:e4965. doi: 10.7717/peerj.4965, PMID: 29888140 PMC5991299

[ref38] Ogbe A. A. Finnie J. F. Van Staden J. (2020). The role of endophytes in secondary metabolites accumulation in medicinal plants under abiotic stress. S. Afr. J. Bot. 134, 126–134. doi: 10.1016/j.sajb.2020.06.023

[ref39] Paz Z. Komon-Zelazowska M. Druzhinina I. S. Aveskamp M. M. Shnaiderman A. Aluma Y. (2010). Diversity and potential antifungal properties of fungi associated with a Mediterranean sponge. Fungal Divers. 42, 17–26. doi: 10.1007/s13225-010-0020-x

[ref40] Pearman W. Freed N. Silander O. (2019). Testing the advantages and disadvantages of short-and long-read eukarytotic metagenomics using simulated reads. BMC Bioinformatics 21, 1–15. doi: 10.1186/s12859-020-3528-4 PMC7257156 32471343

[ref41] Pita L. Rix L. Slaby B. M. Franke A. Hentschel U. (2018). The sponge holobiont in a changing ocean: from microbes to ecosystems. Microbiome 6:46. doi: 10.1186/s40168-018-0428-1, PMID: 29523192 PMC5845141

[ref42] Poonyth A. D. Hyde K. Peerally A. (1999). Intertidal fungi in Mauritian mangroves. Environ. Sci. 42, 243–252. doi: 10.1515/BOT.1999.028

[ref43] Rämä T. Davey M. L. Nordén J. Halvorsen R. Blaalid R. Mathiassen G. H. (2016). Fungi sailing the ArctiC Ocean: speciose communities in North Atlantic driftwood as revealed by high-throughput amplicon sequencing. Microb. Ecol. 72, 295–304. doi: 10.1007/s00248-016-0778-9, PMID: 27147245

[ref44] Saeed A. F. U. H. Su J. Ouyang S. (2021). Marine-derived drugs: recent advances in cancer therapy and immune signaling. Biomed. Pharmacother. 134:111091. doi: 10.1016/j.biopha.2020.111091, PMID: 33341044

[ref45] Sahoo S. Subban K. Chelliah J. (2021). Diversity of marine macro-algicolous endophytic fungi and cytotoxic potential of *Biscogniauxia petrensis* metabolites against cancer cell lines. Front. Microbiol. 12:650177. doi: 10.3389/fmicb.2021.650177, PMID: 34194402 PMC8236939

[ref46] Shen S. Liu D. Wei C. Proksch P. Lin W. (2012). Purpuroines A-J, halogenated alkaloids from the sponge *Iotrochota purpurea* with antibiotic activity and regulation of tyrosine kinases. Bioorg. Med. Chem. 20, 6924–6928. doi: 10.1016/j.bmc.2012.10.014, PMID: 23131412

[ref47] Sibero M. T. Probadi R. Larasati S. J. H. Calabon M. S. Sabdono A. Subagiyo S. (2021). Diversity of sponge-associated fungi from a mangrove forest in Kemujan Island, Karimunjawa National Park, Indonesia. Biodiversitas 22, 5695–5605. doi: 10.13057/biodiv/d221256

[ref48] Song Y. P. Miao F. P. Liang X. R. Yin X. L. Ji N. Y. (2019). Harziane and cadinane terpenoids from the alga-endophytic fungus *Trichoderma asperellum* A-YMD-9-2. Phytochem. Lett. 32, 38–41. doi: 10.1016/j.phytol.2019.05.001

[ref49] Suryanarayanan T. S. (2012). The diversity and importance of fungi associated with marine sponges. Bot. Mar. 55, 553–564. doi: 10.1515/bot-2011-0086

[ref50] Suryanarayanan T. S. Venkatachalam A. Thirunavukkarasu N. Ravishankar J. P. Doble M. Geetha V. (2010). Internal mycobiota of marine macroalgae from the Tamilnadu coast: distribution, diversity and biotechnological potential. Bot. Mar. 53, 457–468. doi: 10.1515/bot.2010.045

[ref51] Turner E. C. (2021). Possible poriferan body fossils in early Neoproterozoic microbial reefs. Nature 596, 87–91. doi: 10.1038/s41586-021-03773-z, PMID: 34321662 PMC8338550

[ref52] Van Soest R. W. M. Boury-Esnault N. Hooper J. N. A. Rützler K. de Voogd N. J. Alvarez B. (2020). World Porifera database. Available at: http://www.marinespecies.org/porifera (Accessed March, 2020).

[ref53] Venkatachalam A. Govinda Rajulu M. B. Thirunavukkarasu N. Suryanarayanan T. S. (2015). Endophytic fungi of marine algae and seagrasses: a novel source of chitin modifying enzymes. Mycosphere 6, 345–355. doi: 10.5943/mycosphere/6/3/10

[ref54] Wainwright B. J. Bauman A. G. Zahn G. L. Todd P. A. Huang D. (2019). Characterization of fungal biodiversity and communities associated with the reef macroalga *Sargassum ilicifolium* reveals fungal community differentiation according to geographic locality and algal structure. Mar. Biodivers. Spring. 49, 2601–2608. doi: 10.1007/s12526-019-00992-6

[ref55] Wang X. Singh P. Gao Z. Zhang X. Johnson Z. I. Wang G. (2014). Distribution and diversity of planktonic fungi in the West Pacific warm pool. PLoS One 9:e101523. doi: 10.1371/journal.pone.0101523 24992154 PMC4081592

[ref56] White T. J. Bruns T. Lee S. Taylor J. W. (1990). “Amplification and direct sequencing of fungal ribosomal RNA genes for phylogenetics” in PCR Protocols: A Guide to Methods and Applications. eds. Innis M. A. Gelfand D. H. Sninsky J. J. White T. J. (New York: Academic Press Inc), 315–322.

[ref57] Wickham H. (2016). Ggplot2: Elegant Graphics for Data Analysis. New York: Springer-Verlag

[ref58] Wong Chin J. M. Puchooa D. Bahorun T. Jeewon R. (2021). Antimicrobial properties of marine fungi from sponges and brown algae of Mauritius. Mycology 12, 231–244. doi: 10.1080/21501203.2021.1895347, PMID: 34900379 PMC8654394

[ref59] Xu W. Gao Y. H. Gong L. F. Li M. Pang K. P. Luo Z. H. (2019). Fungal diversity in the deep-sea hadal sediments of the yap trench by cultivation and high throughput sequencing methods based on ITS rRNA gene. Deep-Sea Res. I Oceanogr. Res. Pap. 145, 125–136. doi: 10.1016/j.dsr.2019.02.001

[ref60] Yang S. Q. Li X. M. Xin L. Li H. L. Meng L. H. Wang B. G. (2018). New citrinin analogues produced by coculture of the marine algal-derived endophytic fungal strains *aspergillus sydowii* EN-534 and *Penicillium citrinum* EN-535. Phytochem. Lett. 25, 191–195. doi: 10.1016/j.phytol.2018.04.023

[ref61] Yu Z. Zhang B. Sun W. Zhang F. Li Z. (2013). Phylogenetically diverse endozoic fungi in the South China Sea sponges and their potential in synthesizing bioactive natural products suggested by PKS gene and cytotoxic analysis. Fungal Divers. 58, 127–141. doi: 10.1007/s13225-012-0192-7

[ref62] Yurchenko A. N. Berdyshev D. V. Smetanina O. F. Ivanets E. V. Zhuravleva O. I. Rasin A. B. (2019). Citriperazines A-D produced by marine algae-derived fungus *Penicillium* sp. KMM 4672. Nat. Prod. Res. 1-6, 1118–1123. doi: 10.1080/14786419.2018.1552696 30663353

[ref63] Zou J. X. Song Y. P. Ji N. Y. (2019). Deoxytrichodermaerin, a harziane lactone from the marine algicolous fungus *Trichoderma longobrachiatum* A-WH-20-2. Nat. Prod. Res., 35, 216–221. doi: 10.1080/14786419.2019.1622110 31140305

