# Supplementary figures and images for: Metabarcoding assessment of fungal diversity in brown algae and sponges of Mauritius
Source: Front Microbiol. 2022 Oct 28;13:1003790. doi: 10.3389/fmicb.2022.1003790 (PMC9649896; doi:10.3389/fmicb.2022.1003790)

Figure S1

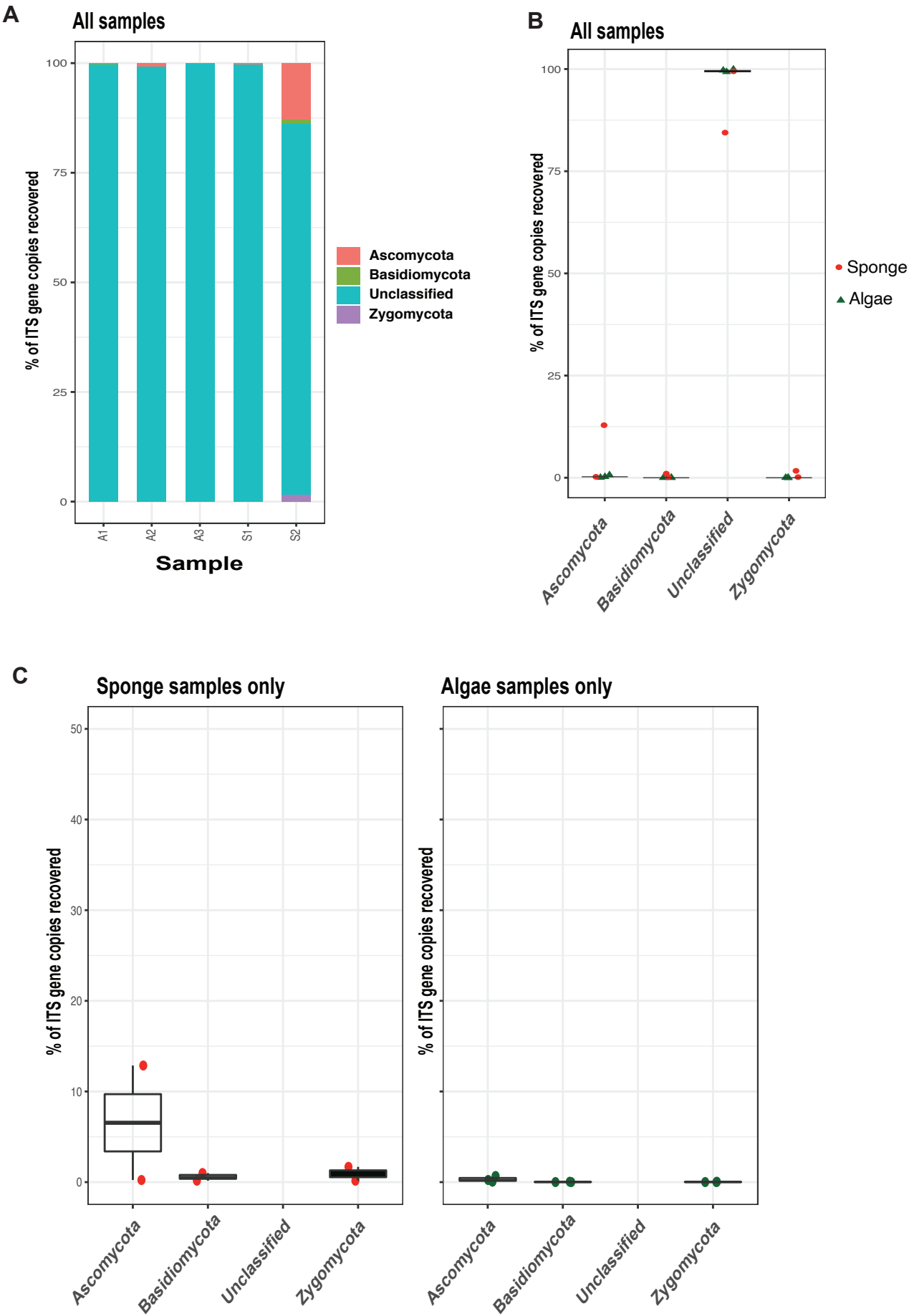

Figure S2

A

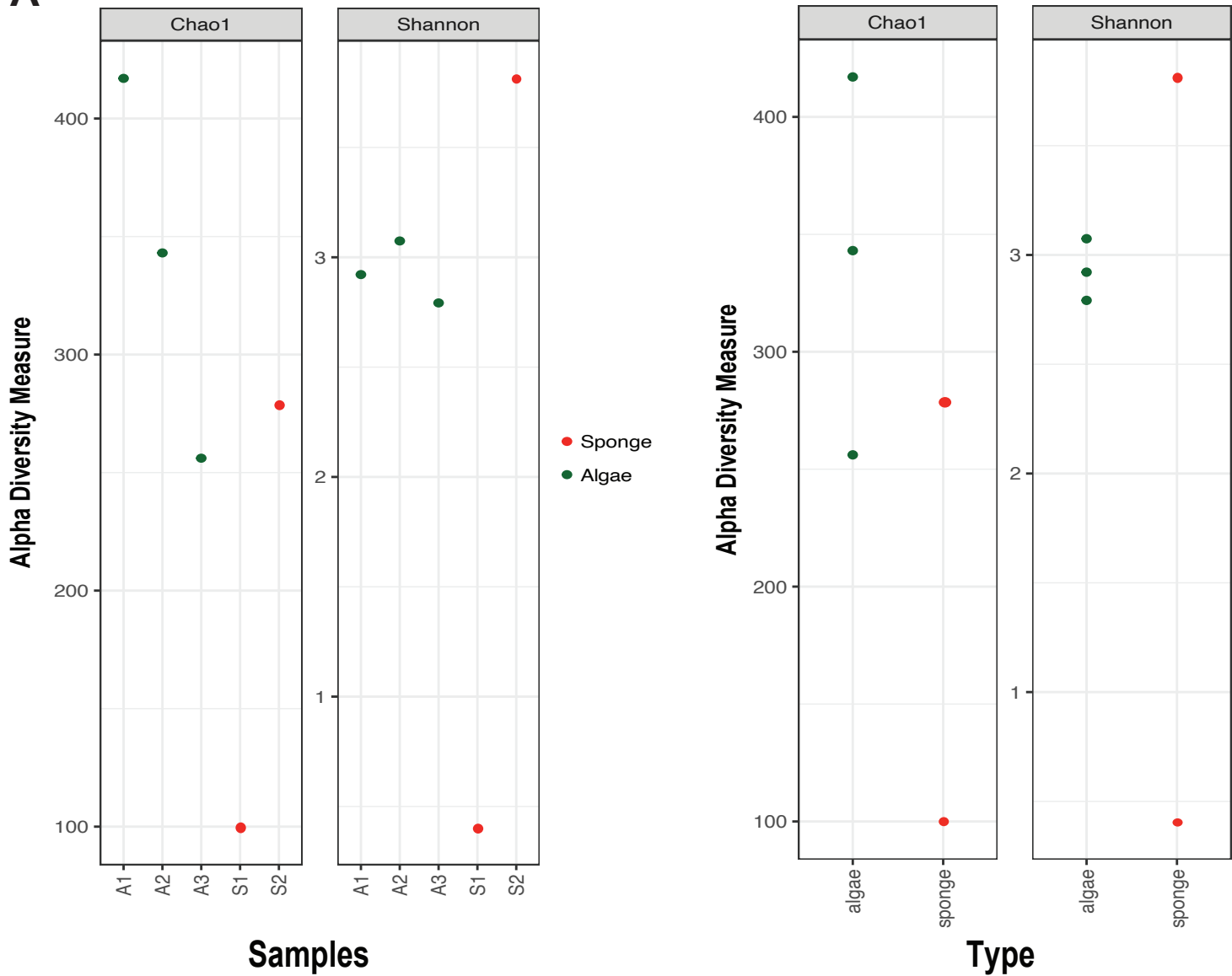

B

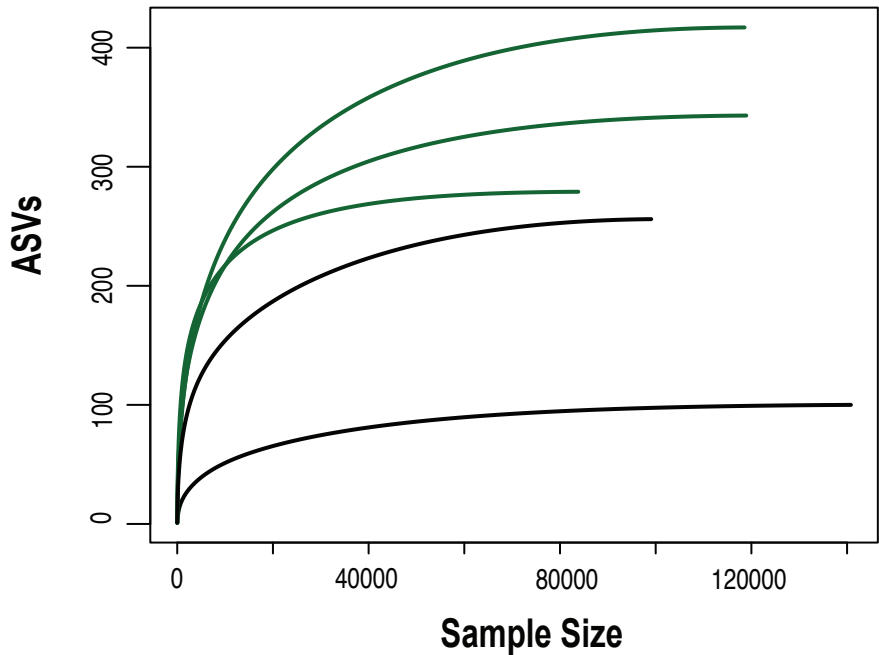

Supplement: Supplementary Figure 1 — Community composition. (A) Taxonomic summary at phylum level in all the samples. (B) Boxplot showing percentage of ITS gene copies recovered in all the samples. (C) Boxplot showing fungal diversity in sponge (red dots) and algae (green dots) separately. [file Data_Sheet_1.PDF]
